# Training the next generation of clinical trial biostatisticians

**DOI:** 10.1177/17407745251361741

**Published:** 2025-08-08

**Authors:** Sameer Parpia, Jacquelyn Dobinson, Anna Heath, Hubert Wong, Kevin Thorpe, Tolulope Sajobi, Shirin Golchi, Lawrence Mbuagbaw, Shun Fu Lee, Thi Ho, Valerie Bishop

**Affiliations:** 1 Department of Oncology, McMaster University, Hamilton, ON, Canada; 2 Health Research Methods, Evidence & Impact, McMaster University, Hamilton, ON, Canada; 3 Child Health Evaluative Sciences, The Hospital for Sick Children, Toronto, ON, Canada; 4 Dalla Lana School of Public Health, University of Toronto, Toronto, ON, Canada; 5 Department of Statistical Science, University College London, London, UK; 6 School of Population and Public Health, University of British Columbia, Vancouver, BC, Canada; 7 Cumming School of Medicine, University of Calgary, Calgary, AB, Canada; 8 School of Population and Global Health, McGill University, Montreal, QC, Canada; 9 Population Health Research Institute, Hamilton, ON, Canada

**Keywords:** Clinical trials, biostatistics, mentorship, training

## Abstract

**Background:**

There is a critical shortage of biostatistics expertise and targeted training programs in clinical trials across Canada.

**Methods:**

The Canadian Network for Statistical Training in Trials (CANSTAT), a pan-Canadian, multi-institutional training platform for biostatisticians in clinical trials, was developed to increase clinical trial biostatistics capacity in Canada.

**Results:**

CANSTAT’s training program integrates experiential learning through mentorship and placements at clinical trial sites, online workshops, and capacity-building meetings. The curriculum is designed to equip fellows with essential knowledge of clinical trials, technical skills, and practical experience necessary for their growth into professional trial biostatisticians, with several specific and measurable objectives set to achieve this goal. Educational materials, including CANSTAT competencies, reflective exercises, and individual development plans, are provided to monitor progress and ensure that fellows are meeting their academic and professional goals. Currently, CANSTAT has enrolled 19 fellows.

**Conclusion:**

CANSTAT has developed a training program that equips fellows with essential skills in clinical trial design, conduct and analysis, and interprofessional communication, preparing them to effectively lead biostatistical efforts in clinical trials. By training a new generation of clinical trial biostatisticians, CANSTAT is strengthening Canada’s clinical trial enterprise and improving health outcomes.

## Background

Biostatisticians play a pivotal role in the design, monitoring, conduct, analysis, reporting, and interpretation of clinical trial outcomes,^1^ collaborating closely with clinical investigators throughout the entire process. Their expertise is essential to ensuring the validity and robustness of trial results, as they are highly influenced by the statistical and methodological approaches employed.^2^ The absence of biostatistical and methodological input at all stages of a trial is a significant contributor to poor-quality research.^3^

Given that clinical trials are a gold standard for generating high-quality evidence on the efficacy, effectiveness, and safety of medical interventions,^4^ the role of biostatisticians becomes even more crucial in ensuring that these trials produce reliable evidence that can shape clinical practice, inform policy, and improve health outcomes. Biostatisticians contribute not only to the scientific rigor of individual studies but also to the broader public health system by supporting the generation of trustworthy evidence that directly impacts patient care.^5^

Despite the growing demand for evidence to inform approval of new medicines, clinical practice, devices, health technologies, surgical techniques, and public health decisions, the critical shortage in the clinical trials workforce has been identified as a significant barrier to the advancement of the clinical trial enterprise. In particular, there is a critical shortage of trial biostatisticians with expertise in the design and analysis of clinical trials to accelerate scientific discoveries in clinical research. In 2010, as part of the Strategy for Patient-Oriented Research, the Canadian Institutes of Health Research (CIHR) highlighted that health science centers involved in clinical trial research commonly report critical shortages of biostatisticians and methodologists.^6^ In addition, a survey of oncologists conducted by the Ontario Institute of Cancer Research (OICR) identified that among 80% of respondents, the lack of trained biostatisticians was a factor limiting their progress in cancer research.^7^ This view is not specific to oncology but is shared among the broader health research community.

Highly qualified personnel in mathematics and (bio)statistics are uniquely suited to fill this shortage in the clinical trials workforce. However, there is a significant gap between the competencies taught in mathematics and (bio)statistics graduate programs compared with those required for work in clinical trials. Mathematics and (bio)statistics graduate programs provide a robust theoretical foundation in statistics; however, they offer limited exposure to the practical aspects of clinical trial design, implementation, monitoring, and analysis.^8^ Similarly, students have limited training in other important aspects of the clinical trials enterprise, such as data management, ethics, and regulatory processes, and essential personal skills including collaboration, communication, and project management, that are required to function as biostatistical leaders in multidisciplinary clinical trial projects.

The Canadian Network for Statistical Training in Trials (CANSTAT) emerged as part of the response to addressing the shortage of clinical trial biostatisticians in Canada. CANSTAT is a pan-Canadian, multi-institutional, and multidisciplinary platform with the objective of training and mentoring highly qualified individuals in clinical trials. This article describes the development of this innovative training program, presents the progress, and discusses its strengths and limitations.

## Methods

### Governance

An essential first step taken by CANSTAT was the establishment of an organizational framework and solidifying collaboration with key stakeholders. A well-established governance structure helps to clarify reporting and decision-making processes as the program matures.^9^ CANSTAT established a governance structure constituting an Executive Committee and Advisory Committee, as well as four subcommittees: Recruitment and Admissions, Education and Workshop, Mentorship and Experiential Learning, and Operations. Each committee has defined terms of reference outlining its purpose, roles and responsibilities, decision-making processes, membership terms, and meeting frequency. The subcommittees regularly report progress, challenges, and discussion points to the Executive Committee and Advisory Committee for approval and guidance.

### Curriculum objectives

Recent approaches to training biostatisticians in collaborative research settings call for interdisciplinary skills—specifically communication, teamwork, and subject matter knowledge—alongside technical proficiency.^10^ In addition, mentorship and statistical advising have been recognized as a “key activity in the biostatistician’s professional development and, with the appropriate frameworks, are beneficial for both the biostatistician and the biomedical research process.”^11^

It was important to ensure that program objectives were aligned with the curriculum^12^ as well as the needs of the fellows, mentors, and industry landscape.^13^ A curriculum that blends theoretical knowledge and practical experience is advantageous to the learners^10^ and provides workload support to those who are supervising. In addition, leveraging both virtual and in-person meetings offers flexibility to learners and facilitators while also providing opportunities for networking and community-building.^14^ Thus, the CANSTAT program was designed to blend statistical theory, practical experiences, and mentorship opportunities.

The curriculum was established to equip fellows with clinical trial knowledge, technical skills, and the practical experience needed to develop into trial biostatistician professionals. Several specific and measurable objectives were created to achieve this outcome:

1. Provide a solid foundation in the fundamentals of clinical trial methodology.
2. Develop a strong biostatistical foundation in the design and analysis of both conventional and innovative clinical trials.
3. Provide fellows with experiential learning opportunities within a clinical trials environment, including:
  a.Participating in the design, conduct, monitoring, analysis, and reporting of clinical trials.
  b.Cultivating skills relevant to all essential biostatistical roles, including, but not limited to, critical appraisal of proposals and manuscripts, preparation of grants and manuscripts, data management, and participation in trial oversight committees.
  c.Developing effective biostatistical communication (oral presentation and written) and collaboration skills.
  d.Interacting with and learning from other professionals involved in the conduct of clinical trials.
4. Build mentor and trainee relationships that offer mutual learning experiences.
5. Promote a collaborative culture among biostatisticians and non-biostatistician researchers.
6. Build a lasting network of fellows and mentors that will raise the quality of statistical practice in clinical trials throughout Canada.

### Admissions

A robust application process was established to ensure that high-caliber fellows were admitted into the program. To be eligible for the program, applicants must have a master’s or doctoral degree in (bio)statistics or a related field within the health sciences discipline and a strong quantitative background. Applicants are considered for the program based on their academic strength, clinical trial motivation, and exposure, as well as fit with the program.

The application process was informed by several strategies described by Joy.^15^ The first strategy to reduce potential bias is for reviewers to be provided with unconscious bias training modules, multiple reviewers to evaluate each application, and standardized scoring rubrics to be utilized. Second, collecting diversity data in the application package to identify any barriers during the admissions process. The third strategy is an active effort to promote diversity among reviewers and candidates.

CANSTAT adopted a holistic review process for applicants, which is considered standard practice in higher education.^16^ A holistic review process involves collecting both qualitative and quantitative data from application packages to obtain “a spectrum of criteria that reflect applicants’ attitude, knowledge, skills, and competencies.”^16^ Documents collected and reviewed by the Admissions Committee include an application form, a statement of interest highlighting research experience and expectations of the program, a curriculum vitae, transcripts, two letters of recommendation, and an optional letter explaining any career disruptions to allow for an equitable assessment of their application. In addition, interviews between mentors and candidates are conducted to ensure a good fit and clarify expectations for all parties involved. This practice is important as it “addresses non-academic attributes, values, and experiences as well as mitigates barriers for the defined underrepresented minority populations.”^15^

### Experiential learning placement

The core feature of the training program is a full-time placement at an organization actively involved in clinical trials, where fellows are co-mentored by both a clinical trial biostatistician and a clinician investigator. The collaboration between biostatisticians and clinicians is fundamental to successful clinical trial development and execution. Clinical mentors provide expertise and insights that enable trainees to understand research goals and recognize the practical challenges and non-statistical considerations that must be navigated throughout the trial process. This hands-on approach aligns with Experiential Learning Theory, which emphasizes that learning is most effective through direct experience.^17^

To maintain consistency and quality, the program has established a set of competencies (Table 1). These competencies, developed within a Competency-Based Education (CBE) framework, define specific learning outcomes for fellows.^18^ Widely used in both industry and higher education, CBE ensures that learners demonstrate mastery of knowledge and skills applicable to real-world settings, creating a more relevant and impactful educational experience that aligns with professional demands.^19^

**Table 1.** CANSTAT experiential learning competencies.^1^

Key concepts	Mandatory competencies Upon completion of the CANSTAT Program, fellows will be able to …
Trial Design	Understand randomized trial design using necessary components of trial design.
Sample Size Estimation/Power Analysis	Estimate an appropriate sample size and power calculations for new clinical trials.
Randomization	Design randomization schemes for ongoing trials.
Statistical Analysis Plan (SAP)	Develop a Statistical Analysis Plan using best practice guidelines.
Data Management	Manage data for analysis as defined in a Statistical Analysis Plan using standardized software.
Data Analysis	Analyze and interpret trial data as described in the Statistical Analysis Plan.
Steering/Executive Committee	Understand the practical aspects of running clinical trials.
Data Safety Monitoring Boards	(1) Understand how to prepare and present statistical reports to Data Safety Monitoring Boards. (2) Understand how interim trial analysis can lead to recommendations on studies.
Communication	Articulate concepts clearly through oral and written communication.
Key concepts	Optional competencies
Regulated Trials	Understand the requirements and complexities of obtaining regulatory approval forclinical trials.
Grant Preparation	Write a sample size description and an analysis plan for a grant application.
Case Report Form Design/Review	Review data collection instruments to ensure that study aims can be achieved.
Manuscript Review	Complete a peer review of a submitted manuscript.

1 Table 1 illustrates the competencies developed by CANSTAT that define specific learning outcomes for fellows.

The CANSTAT competencies were created by a group of biostatistical experts with diverse specializations. The competencies outline the essential knowledge and skills fellows are expected to acquire during their training to prepare them for successful entry into the clinical trials field. Thirteen competencies were identified, nine of which were deemed mandatory, while the remaining four were considered optional. The optional competencies were labeled as such because although they are important, some institutions may not be able to provide those opportunities to the fellows.

During the placement, mentors expose fellows to as many aspects of clinical trials as possible. The mentors collaborate with fellows to create a comprehensive individualized learning plan and meet regularly to review the fellow’s progress. The fellows submit quarterly progress reports to the Mentorship and Experiential Learning Committee, which reviews the reports to ensure that fellows meet the program competencies.

In addition to the individual learning plans, fellows prepare reflective summaries that are submitted every 4 months. In line with Experiential Learning Theory, the reflections promote self-awareness, critical thinking, and problem-solving by asking fellows to identify what they have learned, what barriers they encountered, and how they intend to achieve their goals by the end of the program. In addition, the reflections provide a quality assurance mechanism, as they are a way for issues to be quickly identified and resolved. Likewise, mentors are also asked to complete a reflection at the end of the year to provide feedback on the program.

The clinical trial placement was a complex element to establish due to CANSTAT’s multi-institutional structure. To address this, guidelines were created for both mentors and fellows, aiming to standardize the placements and establish a “contract between the educational institution and the student.”^18^ These guidelines provide clear direction on roles, responsibilities, expectations, and the management of the experiential learning placement. Additional support for mentors, such as annual mentor meetings, has also been implemented to provide an opportunity for program feedback and discussion.

### Online workshops

The second key component of the curriculum is a series of online workshops. Online workshops offer flexibility, encourage self-directed learning, and promote collaboration, all of which contribute to effective skills development.^20,21^ The workshops take place online due to the geographic locations and differing time zones of fellows and instructors. This format allows fellows the flexibility to attend live discussions with their colleagues across Canada. In addition, the workshops support experiential learning by introducing new concepts and theories that fellows can apply to practical problem-solving.

Fellows participate in 18 online workshops and presentations led by a diverse group of subject matter experts. Each workshop has preparatory recorded lectures, readings, and activities that can be completed asynchronously, followed by expert-led synchronous discussion sessions. The workshop leads are provided with an honorarium for content development and delivery. Through these workshops, fellows are exposed to the statistical and non-statistical aspects of clinical trials (Table 2). Subject matter experts carefully design the topics of the workshops to ensure their relevance to the field. These topics are determined through consensus within the CANSTAT Education and Workshop Committee and input from the broader network, allowing fellows to gain exposure to various issues that are deemed necessary by those conducting trials.

**Table 2.** CANSTAT workshops.^1^

Workshop titles	Topics covered
Working with Non-biostatisticians	Reflection on professionalism, teamwork, plain language communication, presentation skills,design thinking, and engaging with patient partners.
Ethics & Regulatory Issues in Trials	Research ethics and regulatory considerations.
Reproducible Research	The importance of statistical programming best practices for reproducible research. Topicswill include hardcoding versus programmatic numbers; technical debt; data management; documentation; and version control.
Randomization	Methods for randomization, stratification, creating randomization lists, validating randomization, why randomization, and importance of blinding.
Role of Biomarkers as Replacement (i.e. Surrogate)Endpoints	Criteria for choosing endpoints, “a correlate does not a surrogate make,” validation ofreplacement (surrogate) endpoints, and accelerated and regular approval process.
Endpoint/Outcome Selection	Statistical considerations when choosing outcomes (binary, ordinal, time-to-event,continuous), dichotomization or quantitative outcomes, clinical relevance, patient-reported outcomes, measurement error, efficiency, and patient partner engagement.
Sample Size and Power	Design type considerations, selecting appropriate effect sizes, expected versus actualpower, use of external data, analytic formulae versus simulation, and software.
Analysis Principles	Analysis principles: SAP, intent-to-treat, ICH E9 Addendum, covariate adjustment,pre-specified versus post-hoc analysis.
Addressing Missing Data	Factors contributing to missing data, approaches to addressing missingness, inherentlimitations of approaches for treating missing data, and the successful prevention of missingdata: illustrations and conclusions.
Statistical Writing and Reporting	Grant applications, reporting guidelines (SPIRIT, CONSORT, and extensions, etc.), andknowledge translation.
Economic Analysis	Health economic models, quality of life assessments, utilities, and incorporation into design.
Safety Issues	Adverse events, CTCAE, analysis of safety data.
Non-Inferiority Designs	Motivating the proper role of non-inferiority trials, the constancy assumption: recognizingits importance and addressing uncertainty about its validity, determining the non-inferioritymargin, and illustrating the successful design and conduct of non-inferiority trials.
Early-Stage Designs	Dose-finding studies, dose-optimization, dose-expansion cohorts, and phases I and II.
Bayesian Design	Bayesian design and analysis of clinical trials.
Adaptive Designs	Rationale, benefits and limitations, platform trials, multi-arm multi-stage designs, andBayesian adaptive designs.
Design and Analysis of Vaccine Trials	Types of designs, outcome measures, interim monitoring, and sample size considerations.
Cluster Randomized andStepped Wedge Trial Designs	Rationale, design considerations, analytic models, and sample size calculation.

1 Table 2 summarizes the key statistical and non-statistical topics covered in the workshops designed for fellows.

### Capacity-building meetings

To complement the training provided through the experiential learning placements and online workshops, fellows participate in two in-person, 2-day capacity-building meetings. Studies demonstrate that in-person meetings promote interpersonal connection and the development of professional networks.^14^ The capacity-building meetings foster interactions among fellows, provide significant networking opportunities, and encourage discussions on topics with differing expert opinions. Meetings consist of presentations by experts in clinical trials, followed by small group discussions. These small groups were strategically formed before the meeting to ensure diverse representation. This pre-assignment provided the fellows with an equal opportunity to contribute and helped maintain audience engagement throughout the session. Topics presented at the capacity-building meetings have included Equity, Diversity, and Inclusion in Clinical Trials, Confirmatory Versus Exploratory Analysis, the Role of Estimands in Clinical Trials, Indigenous Data Governance, Data Monitoring Committees, and Clinician-Statistician Interfaces in Bayesian Trials.

## Results

### Progress, achievements, and impact

CANSTAT was awarded $2.5 million in CIHR funding in the Spring of 2022 as a result of the Clinical Trials Fund competition.^22^ The funding has supported program development, honoraria, operations and equipment, capacity-building meetings, and trainee stipends. The program began development in January 2023 and was quick to launch. The first 8 months of CANSTAT were dedicated to the design and development of the program, as well as forming partnerships with a wide range of institutions. CANSTAT welcomed its first cohort of fellows in September 2023. CANSTAT has partnered with 30 organizations, including 17 institutions participating in clinical trials as well as various clinical trial training platforms, research networks, provincial research organizations, and the Accelerating Clinical Trials Consortium. There are 20 mentor pairings (biostatistician and clinical investigator) with diverse research areas and expertise including oncology, psychology, cardiology, nephrology, endocrinology, infectious diseases, rare diseases, pediatric health, and mental health.

To date, 19 fellows have either completed the fellowship or are currently enrolled in the program. Each fellow has been stationed at a clinical trial unit under the guidance of one of the 20 mentor pairs. Fellows have contributed to 77 clinical trials across five Canadian provinces. Their involvement includes grant preparation, trial design, statistical analysis plan development, sample sizes and power estimation, analysis, attendance of steering/executive and data safety monitoring board meetings, and manuscript preparation.

## Discussion

### Looking ahead

As CANSTAT is a newly established platform, considerable effort has gone into developing partnerships, resources, and materials. The connections made thus far have been vital in fostering multi-institutional collaboration and ensuring the successful pan-Canadian implementation of a high-quality training program. Although the program operates within academic and hospital environments, it aims to provide trainees with fundamental skills that prepare them for successful careers across all sectors, whether in academia, government, or industry.

The geographical dispersion of stakeholders across various time zones, as well as the ongoing development of standardized processes, provided opportunities to optimize operational strategies. Virtual meetings and pre-recorded sessions have facilitated the delivery of content without the need for travel. Course content has been delivered virtually via Raising Interdisciplinary Scientist Excellence (RISE),^23^ a learning management system created by Empowering Next-Generation Researchers in Perinatal and Child Health (ENRICH). Although the geographical dispersion created some challenges, it also ensured that the program could operate on a national scale and enabled CANSTAT representation across most of the country.

Knowledge and best practices within the clinical trial industry continue to evolve, and information changes rapidly. Hence, CANSTAT performs an annual review of the curriculum to confirm learning materials and information are contemporary and relevant to the clinical trials industry.

Upon completion of the fellowship, CANSTAT fellows and mentors complete comprehensive evaluations assessing their experience and identifying areas for improvement, with fellows specifically rating their confidence in independent clinical trial work, likelihood to recommend the program, and how effectively the program delivered competency-based training, fostered meaningful mentor–trainee relationships, and strengthened their biostatistical expertise in conventional and innovative trial design and analysis. In addition, 1-year follow-up surveys will be conducted to track fellows’ career trajectories, determine their current involvement in clinical trials or other work, assess which CANSTAT competencies they apply in practice, and evaluate how well the program prepared them for their professional roles.

Securing sustainable funding beyond the initial 3-year period remains a critical priority for the network. Funds received to date have been leveraged creatively, and a variety of complementary funding models and program adaptations are being considered to achieve program longevity. This includes co-funding partnerships with provinces and institutions, offering fee-for-service options, building sponsorship opportunities with industry partners, and submitting applications to fund-granting foundations. Future programs should secure long-term commitments from supporting organizations before launching to ensure sustainability.

## Conclusion

To address the critical shortage of clinical trial biostatisticians in Canada, CANSTAT has developed a training and mentoring program that combines experiential learning, online workshops, and capacity-building meetings. Through this process, CANSTAT has built a community of biostatistician and methodologist experts, which enhances multi-institutional collaboration and innovation within clinical trials.

The program’s design intentionally responds to the evolving demands of biostatisticians and methodologists working in clinical trials. The curriculum aligns with contemporary best practices for training future leaders in the field. The program’s success relies on its structure and the mechanisms that support delivery, including its recruitment strategies, technical training, mentorship framework, and experiential learning opportunities. By improving the design, conduct, and analysis of clinical trials, the program aims to generate high-quality evidence that will enhance patient outcomes in Canada.
